# Clinical and prognostic significance of high-endothelial venule density in regional lymph nodes of esophageal adenocarcinoma

**DOI:** 10.3389/fonc.2026.1802849

**Published:** 2026-07-14

**Authors:** Aryan Mirzaie-Kuzehgarani, Tillmann Bedau, Johanna Teloh-Benger, Thomas Zander, Hans Anton Schlößer, Reinhard Büttner, Christiane Bruns, Alexander Quaas

**Affiliations:** 1 Institute of Pathology, Faculty of Medicine and University Hospital Cologne, Cologne, Germany; 2 Center for Integrated Oncology Cologne, University Hospital Cologne, Cologne, Germany; 3 Department of Internal Medicine, Faculty of Medicine and University Hospital Cologne, Cologne, Germany; 4 Department of General, Visceral and Cancer Surgery, Faculty of Medicine and University Hospital of Cologne, Cologne, Germany

**Keywords:** esophageal cancer, high-endothelial venule (HEV), MECA, neoadjuvant treatment, prognosis

## Abstract

**Background:**

Esophageal adenocarcinoma (EAC) is associated with poor prognosis despite advances in multimodal treatment. High endothelial venules (HEVs) facilitate lymphocyte trafficking and are linked to anti-tumor immunity. While HEVs have shown prognostic relevance in esophageal squamous cell carcinoma and gastric cancer, their role in regional lymph nodes of EAC patients remains unclear.

**Methods:**

This retrospective study included 199 patients with EAC who underwent Ivor-Lewis esophagectomy between 2013 and 2021. Patients received perioperative chemotherapy (FLOT), neoadjuvant chemoradiotherapy (CROSS), or primary surgery. Tumor-free regional lymph nodes were immunohistochemically stained using the MECA-79 antibody to detect HEVs. Whole-slide images were digitally analyzed to quantify HEV density. Associations with clinicopathological parameters, treatment modality, therapy response, and survival outcomes were assessed. An exploratory analysis was performed in an additional cohort of 58 patients receiving adjuvant nivolumab therapy.

**Results:**

HEVs were detectable in regional lymph nodes across all treatment groups. HEV density was significantly higher in patients older than 60 years compared to younger patients (p = 0.001) and was independent of treatment modality, pathological response, T-stage, and N-stage. No association was observed between HEV density and response to neoadjuvant therapy. Dichotomization into MECA-low and MECA-high groups confirmed age as the primary determinant of HEV density classification. Kaplan–Meier analyses showed no significant differences in overall or progression-free survival between MECA-low and MECA-high patients in the primary cohort. In the adjuvant nivolumab cohort, MECA-high patients demonstrated a non-significant trend toward improved two-year overall survival (90% vs. 67%).

**Conclusions:**

HEV density in tumor-free regional lymph nodes of EAC patients is primarily age-dependent and independent of tumor stage, treatment response, and neoadjuvant therapy regimen. Although HEV density alone does not predict survival, observed trends in immunotherapy-treated patients suggest a potential modulatory role in immune responsiveness. These findings emphasize the importance of host-related immune architecture and support further investigation of lymph node–based immune biomarkers in EAC.

## Introduction

Esophageal cancer ranks as the 11th most prevalent cancer globally and the 7th leading cause of cancer-related mortality, accounting for approximately 510,000 new cases and 445,000 deaths in 2022 (5). While esophageal squamous cell carcinoma (ESCC) remains the predominant histological subtype worldwide, esophageal adenocarcinoma (EAC) has emerged as the dominant form in North America, Northern Europe, and Oceania due to rising incidence rates in these regions over recent decades (12). Despite recent advances in multimodal treatment approaches, including the establishment of perioperative FLOT chemotherapy as the preferred standard of care for resectable locally advanced EAC with improved 3-year overall survival of 57.4% compared to the CROSS chemoradiotherapy regimen (6), outcomes remain suboptimal for many patients. This underscores the critical need for novel diagnostic biomarkers and therapeutic strategies to further improve patient outcomes in EAC.

High endothelial venules (HEVs) are specialized postcapillary venules that serve as the primary gateways for lymphocyte trafficking and antigen presentation into lymph nodes and other secondary lymphoid organs. HEVs facilitate the transmigration of naive and memory lymphocytes through their thick glycocalyx coat, which is routinely detected using the MECA-79 antibody (3). Beyond their homeostatic role in lymphoid organs, HEVs can form *de novo* within human tumors (TU-HEV), where they contribute to the generation of tertiary lymphoid structures that are commonly associated with favorable prognosis and enhanced anti-tumor immunity (15). TU-HEVs predominantly arise at the tumor periphery or in peritumoral areas and can be induced through various immunostimulatory therapies, including immune checkpoint blockade and anti-angiogenic treatments (15). These structures represent a promising therapeutic target for overcoming T cell exclusion from the tumor microenvironment and converting immunologically ‘cold’ tumors into immune cell-enriched ‘hot’ tumors.

Clinical studies have begun to elucidate the prognostic significance of HEVs in esophageal cancer. Analysis of HEV density using the MECA-79 marker in 52 ESCC patients revealed that TU-HEV presence was significantly associated with improved overall survival, with TU-HEVs predominantly localized within tertiary lymphoid structures and surrounded by abundant lymphocyte populations (9). Similarly, in gastric cancer, which shares anatomical proximity and treatment approaches with EAC, HEVs were identified in 38.2% of cases and demonstrated significant association with increased CD8+ tumor-infiltrating lymphocytes. Patients with high CD8+/HEV+ levels showed the longest overall survival and this combination served as an independent prognostic factor (7, 14). These findings suggest that HEVs may serve as biomarkers for anti-tumor immunity in upper gastrointestinal cancers, though their specific role in esophageal adenocarcinoma remains to be fully characterized.

Despite emerging evidence demonstrating the prognostic significance of HEVs in ESCC and gastric cancer, their specific role and clinical relevance in esophageal adenocarcinoma remains unexplored. Given the distinct epidemiological patterns, molecular characteristics, and treatment responses between EAC and ESCC, findings from squamous cell carcinoma studies may not be directly applicable to adenocarcinoma. Furthermore, while previous studies have focused primarily on HEVs within the primary tumor, the presence and significance of HEVs in regional lymph nodes—critical sites for immune priming and metastatic spread in EAC—have not been systematically investigated. This study aims to characterize HEV presence in regional lymph nodes from EAC surgical resection specimens and explore their association with clinicopathological features, impact of neoadjuvant (radio-chemotherapy), and patient prognosis.

## Materials and methods

### Patient selection and study design

A total of 199 patients with EAC who underwent Ivor-Lewis esophagectomy (resection of the distal esophagus and proximal stomach) at the Department of General, Visceral, Thoracic and Transplant Surgery, University Hospital Cologne, between 2013 and 2021, were included in this retrospective study. All cases were routinely diagnosed at the Institute for Pathology, University Hospital Cologne. Patients were stratified into three treatment groups: those who received perioperative chemotherapy (FLOT) (n=64), those who received neoadjuvant chemoradiotherapy (CROSS) (n=98), and those who underwent primary surgical resection (n=37) without prior (radio-)chemotherapy. This study was conducted in accordance with the Declaration of Helsinki and approved by the Ethics Committee of the Medical Faculty of the University of Cologne (approval number: 24-1089).

### Tissue selection and preparation

Cases were worked up according to routine pathological standards, and a representative slide containing lymph node (LN) tissue without tumor infiltration was selected by a board-certified pathologist specialized in upper gastrointestinal pathology for further analysis in this study. Special attention was paid to exclude (mediastinal) LNs showing signs of anthracosis, as carbon deposits could potentially interfere with subsequent immunohistochemical evaluation. All selected LNs were formalin-fixed and paraffin-embedded (FFPE) following standard histopathological protocols.

### Immunohistochemical staining

Immunohistochemical staining was performed on 3-4 μm thick sections from the selected LNs using the MECA-79 antibody (Santa Cruz, sc-19602; dilution: 1:400) which specifically recognizes HEV. Staining was carried out according to standard immunohistochemical procedures using the Leica Bond stainer (Wetzlar, Germany).

### Digital image analysis

All immunohistochemically stained slides were digitized using a whole slide scanner (Leica Aperio GT 450 DX). The resulting whole slide images were imported into QuPath (version 0.5.1) for quantitative analysis. Regions of interest (ROI) were manually annotated around the LN tissue to define the areas for subsequent analysis.

Within QuPath, the “positive cell detection” classifier was employed to automatically detect all cells within the specified ROI. Cells were classified as positive or negative based on DAB (3,3’-diaminobenzidine) staining intensity, with DAB-positive cells considered MECA-79 positive. Quantitative measurements, including positive and negative cell counts and the number of positive cells/mm^2^, were subsequently exported from QuPath for further statistical analysis.

### Statistical analysis

All measurement data exported from QuPath were imported into R statistical software (version 4.4.3) and analyzed using the tidyverse package collection. Continuous variables are presented as median with interquartile range or mean with standard deviation as appropriate, and categorical variables as frequencies and percentages. Patients were stratified into MECA-high and MECA-low groups based on median MECA density as the cutoff value. Between-group comparisons used Wilcoxon rank-sum tests for two groups and Kruskal-Wallis tests for multiple groups, followed by *post-hoc* pairwise Wilcoxon tests with Benjamini-Hochberg correction when significant. Categorical variables were analyzed using Fisher’s exact test with simulated p-values when appropriate. Overall survival was defined as time from diagnosis to death, and progression-free survival as time to disease progression or death. Survival curves were generated using the Kaplan-Meier method and compared using log-rank tests. Two-year survival rates were calculated with exact binomial confidence intervals. All tests were two-sided with p ≤ 0.05 considered significant. Analyses used R packages: gtsummary, survival, ggsurvfit, rstatix, ggplot2, ggbeeswarm, patchwork, and binom.

## Results

### Patient characteristics

A total of 199 patients with esophageal adenocarcinoma (EAC) were included in this study. The majority were male (n=171, 85.9%) with a mean age of 64.2 ± 10.1 years. Patients received perioperative FLOT (n=64, 32.2%), neoadjuvant CROSS (n=98, 49.2%), or primary surgery (n=37, 18.6%). Significant age differences were observed between treatment groups (p<0.001), with primary surgery patients being older (72.1 ± 9.0 years) compared to FLOT (63.3 ± 9.2 years) or CROSS (61.8 ± 9.5 years) patients. Sex distribution was similar across groups (p>0.9).

Among 162 patients receiving neoadjuvant therapy, pathological response was complete in 58 (35.8%), major in 5 (3.1%), and minor in 99 (61.1%), with no significant difference between FLOT and CROSS (p=0.12). Of the 58 complete local responders, 57 achieved ypT0N0 while one had residual nodal disease (ypT0N1).

Pathological tumor stage differed significantly between groups (p<0.001). Among neoadjuvant patients, complete tumor response (T0) was more frequent with CROSS (42.9%) than FLOT (26.6%). T3 tumors predominated among primary surgery patients (51.4%). Nodal stage distribution was similar across groups (p=0.4), with 56.8% overall having N0 disease. Detailed patient characteristics are summarized in **Table 1**.

**Table 1:** Baseline characteristics of patients with EAC by treatment modality.

Characteristic	Overall (N = 199)	FLOT (N = 64)	CROSS (N = 98)	Primary surgery (N = 37)	p-value
Age at surgery — mean ± SD	64.2 ± 10.1	63.3 ± 9.2	61.8 ± 9.5	72.1 ± 9.0	<0.001
Sex — no. (%)					>0.9
Male	171 (85.9)	54 (84.4)	85 (86.7)	32 (86.5)	
Female	28 (14.1)	10 (15.6)	13 (13.3)	5 (13.5)	
Therapy response — no. (%)					0.12
Full local	58 (35.8)	17 (26.6)	41 (41.8)	–	
Major	5 (3.1)	2 (3.1)	3 (3.1)	–	
Minor	99 (61.1)	45 (70.3)	54 (55.1)	–	
Tumor stage (T) — no. (%)					<0.001
T0	59 (29.6)	17 (26.6)	42 (42.9)	0 (0.0)	
T1	21 (10.6)	6 (9.4)	4 (4.1)	11 (29.7)	
T2	22 (11.1)	6 (9.4)	11 (11.2)	5 (13.5)	
T3	91 (45.7)	32 (50.0)	40 (40.8)	19 (51.4)	
T4	6 (3.0)	3 (4.7)	1 (1.0)	2 (5.4)	
Nodal stage (N) — no. (%)					0.4
N0	113 (56.8)	34 (53.1)	59 (60.2)	20 (54.1)	
N1	39 (19.6)	10 (15.6)	19 (19.4)	10 (27.0)	
N2	30 (15.1)	11 (17.2)	15 (15.3)	4 (10.8)	
N3	18 (8.5)	9 (14.1)	5 (5.1)	3 (8.1)	

Statistical comparisons performed using Fisher’s exact test (categorical variables) and one-way ANOVA (continuous variables).

### Methodological approach and HEV detection

**Figure 1:**
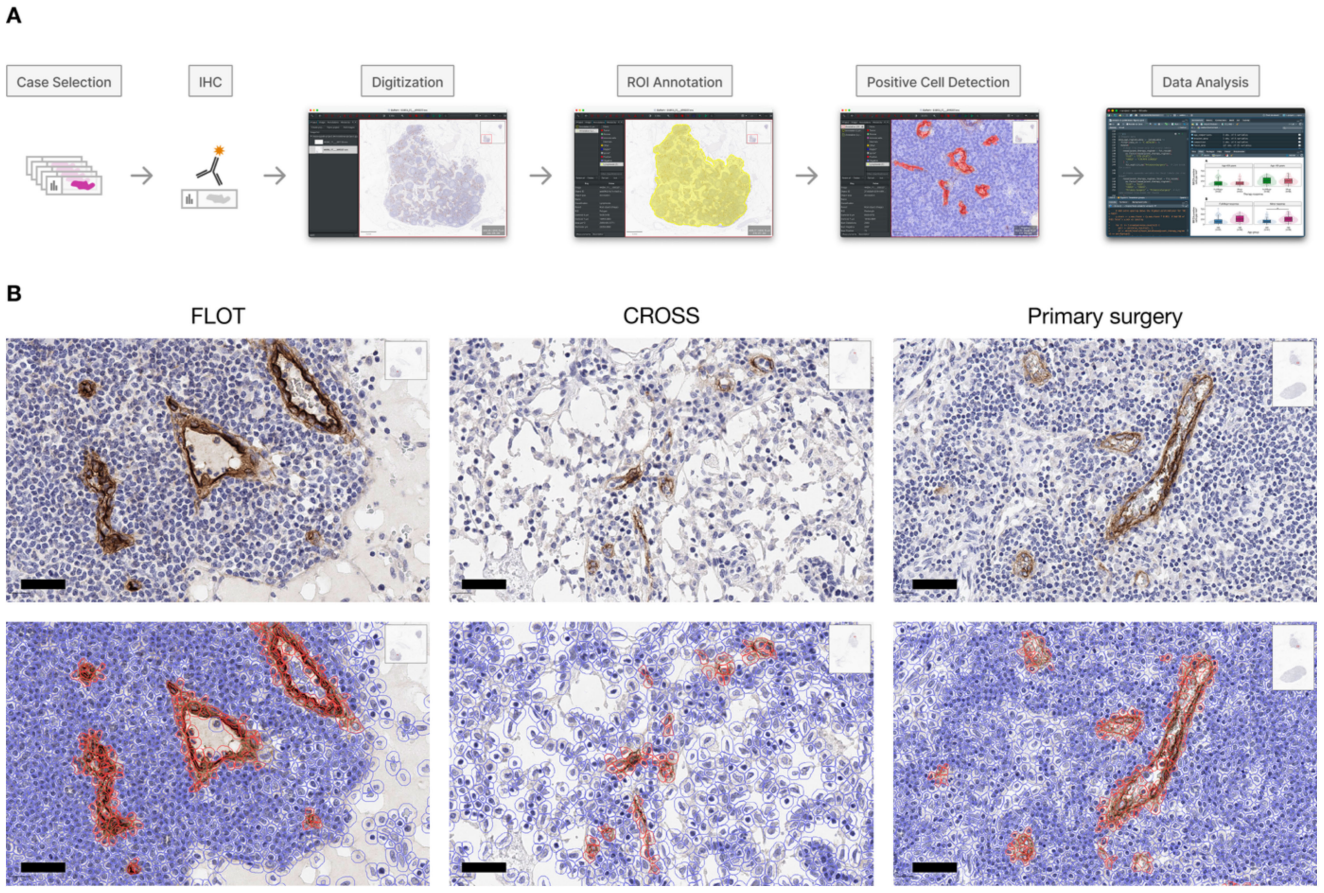
Methodological approach for HEV quantification. **(A)** Analytical pipeline from case selection through immunohistochemistry (IHC), whole-slide digitization, region of interest (ROI) annotation in QuPath (yellow), automated positive cell detection of MECA-positive cells (red), and statistical analysis. **(B)** High-magnification views of HEV across treatment groups (upper row) and corresponding QuPath positive cell detection overlay (lower row, red cells); scale bars = 40 μm.

Our analytical pipeline for quantifying HEV in regional LN is illustrated in **Figure 1A**. Briefly, MECA-79–stained lymph nodes were digitized as whole-slide images, regions of interest around lymph nodes were manually annotated in QuPath, MECA-79 positive cells were quantified by automated detection, and cell counts were exported for statistical analysis. High-magnification views revealed distinct MECA-79 positive HEV across all three treatment groups (**Figure 1B**, upper row). Automated positive cell detection using QuPath successfully identified and quantified MECA-79 positive cells, as demonstrated by the overlay of detected positive cells (red) on the corresponding tissue sections (**Figure 1B**, lower row). This approach enabled systematic and reproducible quantification of HEV density across the entire patient cohort.

### Age-dependent differences in HEV density across treatment groups

**Figure 2:**
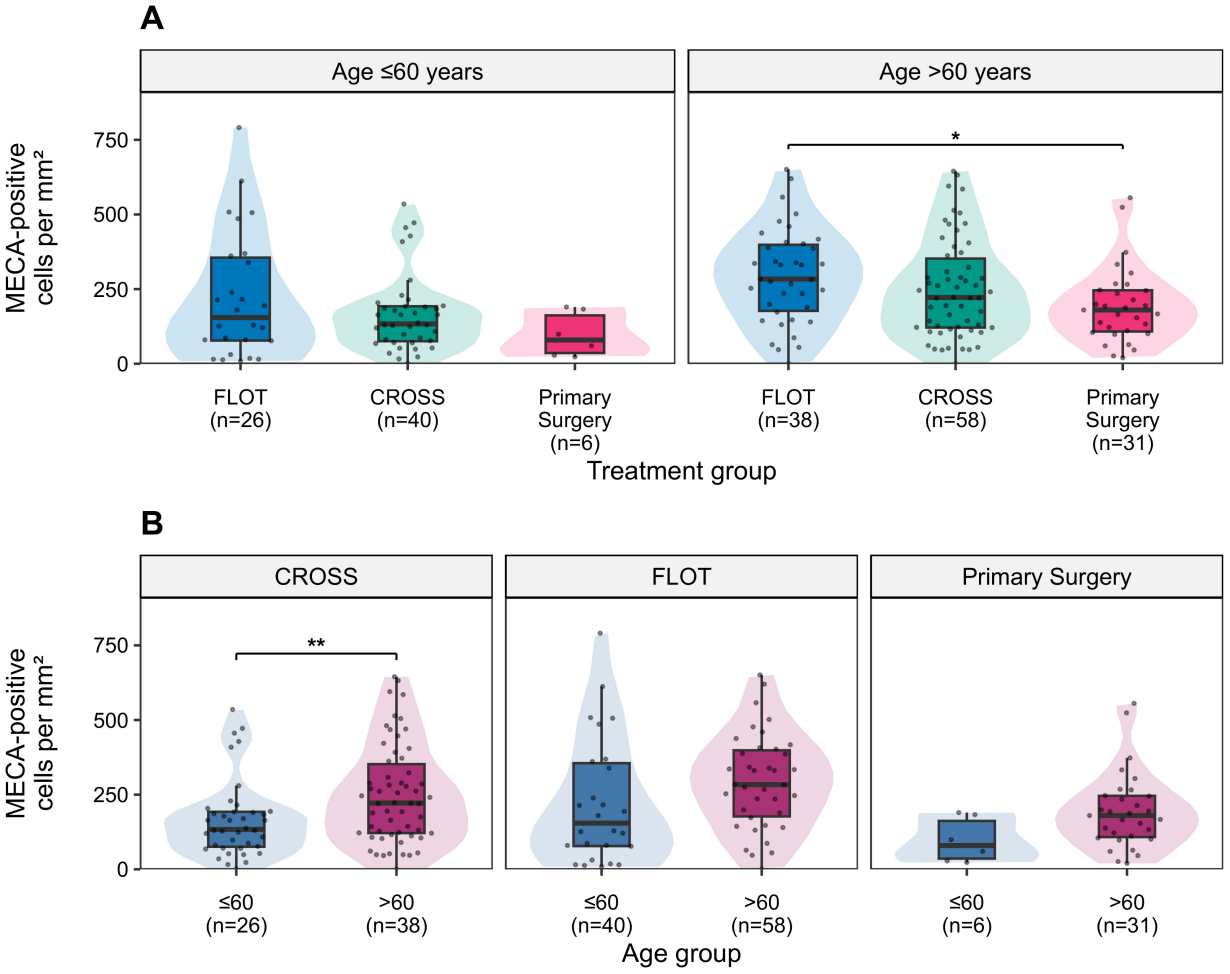
HEV density stratified by treatment modality and age groups. **(A)** Treatment group comparisons within age strata (≤60 years and >60 years). **(B)** Age group comparisons within treatment modalities (CROSS, FLOT, Primary Surgery). Data presented as violin plots with boxplots and individual data points. Statistical significance determined by Kruskal-Wallis test with pairwise Wilcoxon tests **(A)** and Wilcoxon rank-sum tests **(B)**. *p<0.05, **p<0.01.

Building on the established methodology, HEV density analysis revealed distinct patterns dependent on both treatment modality and patient age (**Figure 2**). When stratified by age groups, no significant differences in HEV density were observed between treatment groups in patients ≤60 years (p=0.393). However, in patients >60 years, significant differences emerged between treatment groups (p=0.0204), with *post-hoc* analysis revealing significantly higher HEV density in FLOT-treated patients compared to primary surgery patients (p=0.013).

The most striking finding was the consistent age-related differences within treatment groups. Older patients (>60 years) had significantly higher HEV densities than younger patients (≤60 years) in the CROSS group (p=0.0072). Similar trends were observed in FLOT (p=0.0506) and primary surgery (p=0.0528) groups, although these did not reach statistical significance. Median HEV densities ranged from 79.5 MECA-positive cells/mm² in younger primary surgery patients to 284.0 MECA-positive cells/mm² in older FLOT patients.

### HEV density is independent of therapy response

**Figure 3:**
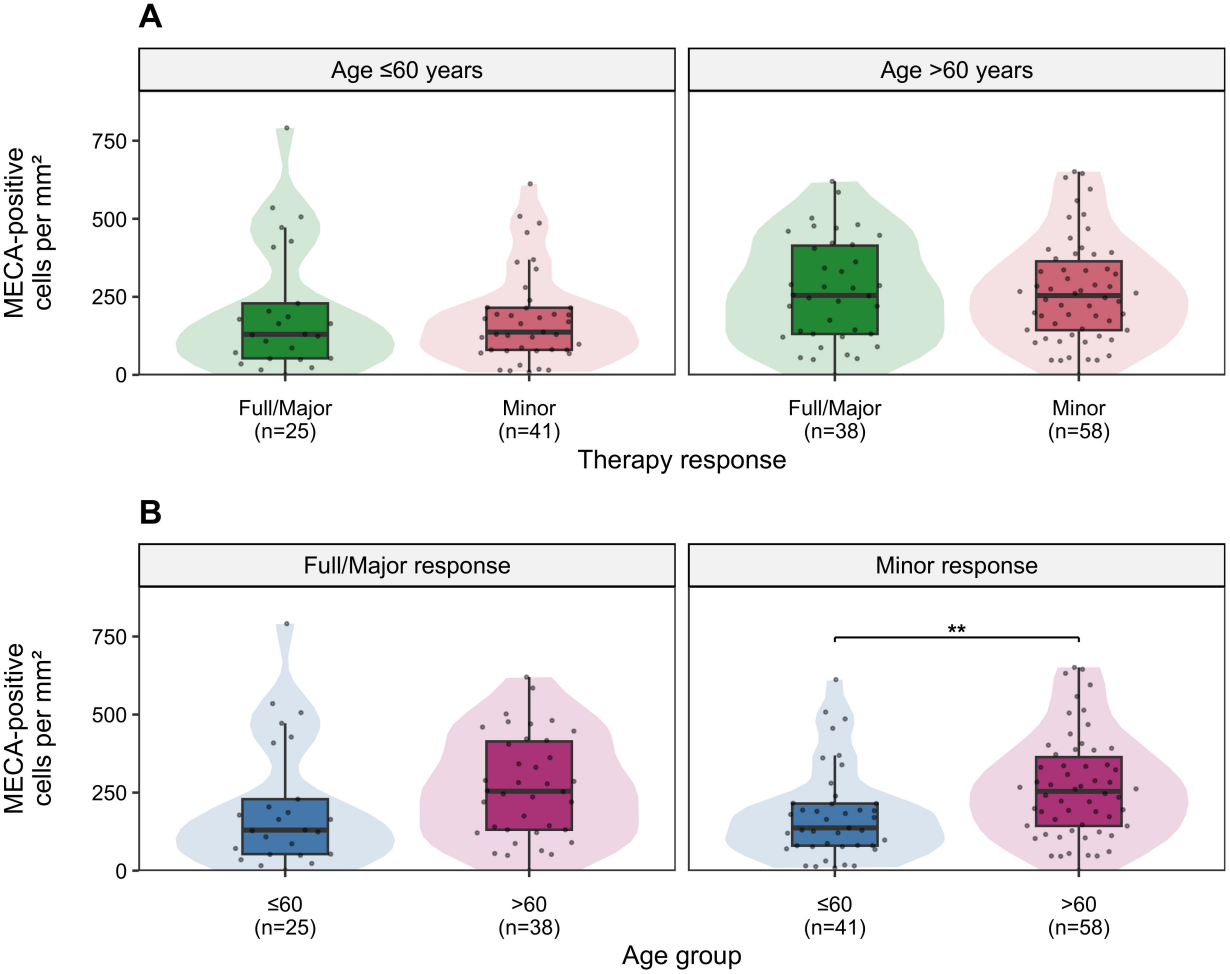
HEV density stratified by therapy response and age groups in neoadjuvant therapy patients. **(A)** Therapy response comparisons within age strata (≤60 years and >60 years). **(B)** Age group comparisons within therapy response categories (Full/Major and Minor). Data presented as violin plots with boxplots and individual data points. Statistical significance determined by Wilcoxon rank-sum tests. **p<0.01.

Given the age-dependent patterns observed with treatment modality, we next examined whether HEV density correlated with therapeutic efficacy prior to surgery. Analysis of HEV density in relation to therapy response was performed in 162 patients who received neoadjuvant therapy (**Figure 3**). Among these patients, 63 (38.9%) achieved a full or major response while 99 (61.1%) had a minor response. Notably, no significant differences in HEV density were observed between therapy response groups in either age stratum (≤60 years: p=0.953, >60 years: p=0.875; **Figure 3A**).

However, the age-related pattern persisted within therapy response groups (**Figure 3B**). Among patients with minor response, older patients (>60 years) demonstrated significantly higher HEV densities compared to younger patients (≤60 years) (p=0.0035). A similar trend was observed in patients with full/major response (p=0.0588), although this did not reach statistical significance. Median HEV densities were consistently higher in older patients regardless of response to neoadjuvant therapy, ranging from 130 MECA-positive cells/mm² in younger patients with full/major response to 254 MECA-positive cells/mm² in older patients with minor response. Of note, no significant associations were observed between HEV density and TNM staging parameters (T-stage: p=0.981, N-stage: p=0.93; **Supplementary Figure 1**).

### Age is the primary determinant of HEV density classification

To further characterize the clinical relevance of HEV density and validate our age-related findings, patients were dichotomized into MECA-low and MECA-high groups using the median HEV density (190.0 MECA-positive cells/mm²) as the cutoff (**Table 2**). This stratification resulted in 99 patients (49.7%) in the MECA-low group and 100 patients (50.3%) in the MECA-high group.

**Table 2:** Patient characteristics stratified by HEV density groups.

Characteristic	Overall (N = 199)	MECA-low (N = 99)	MECA-high (N = 100)	p-value
MECA density				
MECA^+^ cells/mm^2^ (IQR)	190.0 (103.0, 331.0)	103.0 (52.0, 138.0)	327.0 (240.0, 451.5)	<0.001
MECA^+^ cells/all cells (IQR)	1.6 (0.9, 2.6)	0.9 (0.5, 1.2)	2.6 (2.0, 3.7)	<0.001
Treatment regimen — no. (%)				0.095
FLOT	64 (32.2)	25 (25.3)	39 (39.0)	
CROSS	98 (49.2)	52 (52.5)	46 (46.0)	
Primary surgery	37 (18.6)	22 (22.2)	15 (15.0)	
Age group — no. (%)				0.001
≤60 years	72 (36.2)	47 (47.5)	25 (25.0)	
>60 years	127 (63.8)	52 (52.5)	75 (75.0)	
Therapy response — no. (%)				0.7
Full/major	63 (38.9)	31 (40.3)	32 (37.6)	
Minor	99 (61.1)	46 (59.7)	53 (62.4)	
Missing (primary surgery)	37	22	15	
Sex — no. (%)				>0.9
Male	171 (85.9)	85 (85.9)	86 (86.0)	
Female	28 (14.1)	14 (14.1)	14 (14.0)	
T-stage — no. (%)				>0.9
T0	59 (29.6)	29 (29.3)	30 (30.0)	
T1	21 (10.6)	10 (10.1)	11 (11.0)	
T2	22 (11.1)	10 (10.1)	12 (12.0)	
T3	91 (45.7)	47 (47.5)	44 (44.0)	
T4	6 (3.0)	3 (3.0)	3 (3.0)	
N-stage — no. (%)				0.8
N0	113 (56.8)	53 (53.5)	60 (60.0)	
N1	39 (19.6)	21 (21.2)	18 (18.0)	
N2	30 (15.1)	16 (16.2)	14 (14.0)	
N3	17 (8.5)	9 (9.1)	8 (8.0)	

Patients were dichotomized using the median HEV density (190.0 cells/mm²) as cutoff into MECA-low and MECA-high groups. Statistical comparisons performed using Wilcoxon rank-sum test for continuous variables and Fisher’s exact test for categorical variables.

The dichotomization confirmed age as the primary determinant of HEV density classification, with significant differences observed between MECA groups regarding patient age (p=0.001). Older patients (>60 years) were predominantly classified as MECA-high (75.0%) compared to younger patients (25.0%). In contrast, no significant associations were found between MECA groups and treatment regimen (p=0.095), sex (p>0.9), therapy response (p=0.7), T-stage (p>0.9), or N-stage (p=0.8). The median MECA density was 103.0 MECA-positive cells/mm² (IQR: 52.0-138.0) in the MECA-low group and 327.0 MECA-positive cells/mm² (IQR: 240.0-451.5) in the MECA-high group (p<0.001).

### HEV density does not predict overall survival

**Figure 4:**
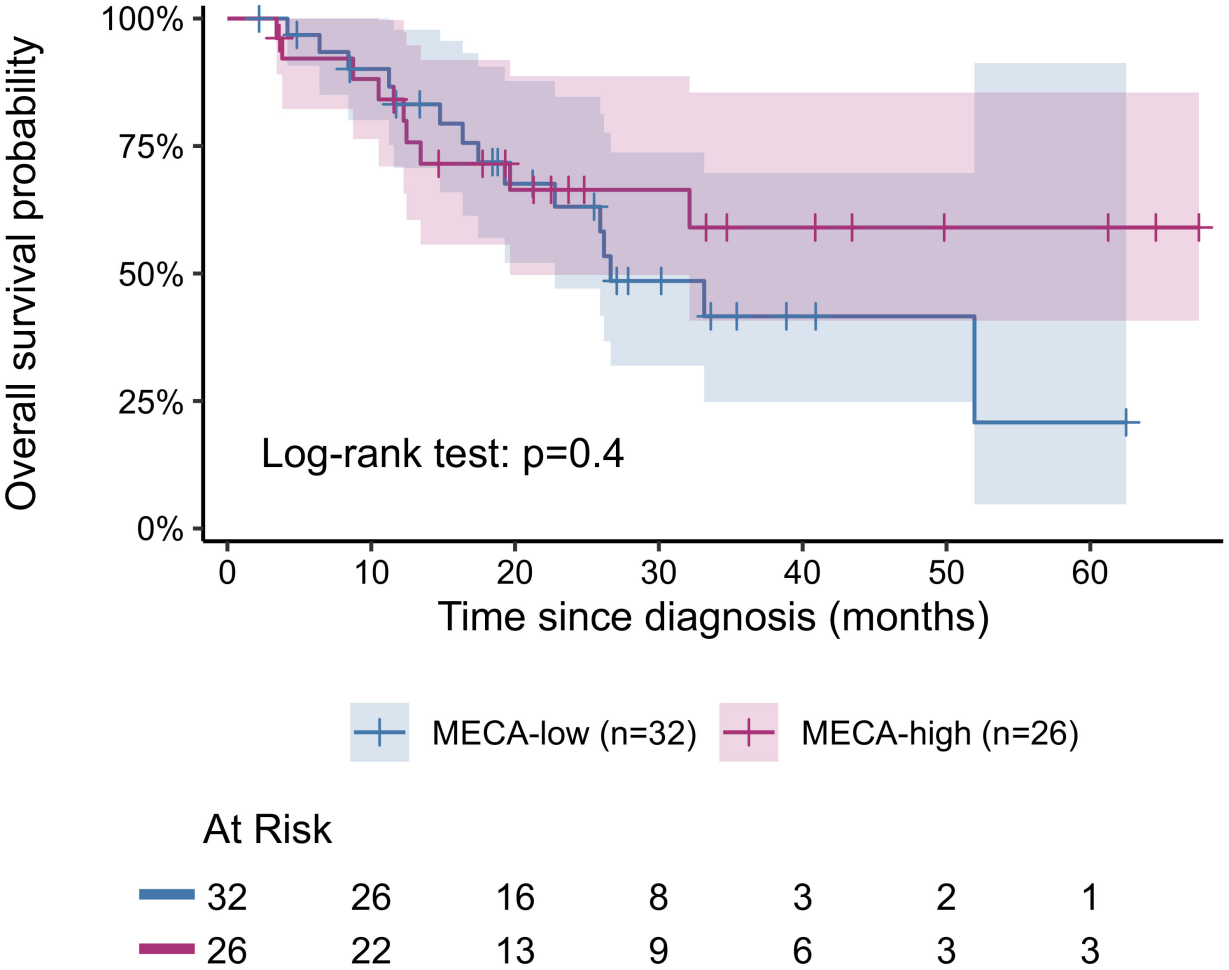
Kaplan-Meier survival analysis stratified by HEV density groups. Overall survival curves for MECA-low (n=32) and MECA-high (n=26) groups with 95% confidence intervals. Numbers at risk are displayed below the plot at each time point. Statistical comparison performed using log-rank test (p=0.4).

We assessed whether the observed differences in HEV density translate into prognostic significance. Kaplan-Meier survival analysis was performed to assess the prognostic value of HEV density stratification (**Figure 4**). Among patients with available survival data, 32 patients were classified as MECA-low and 26 patients as MECA-high. The log-rank test revealed no significant difference in overall survival between MECA-low and MECA-high groups (p=0.4). Both groups demonstrated similar survival trajectories over the follow-up period, indicating that while HEV density shows clear age-related patterns, it does not serve as an independent prognostic indicator for overall survival in this cohort of EAC patients.

### HEV density as a potential biomarker for adjuvant immunotherapy response

To explore the potential clinical utility of HEV density as a prognostic biomarker in the context of immunotherapy, we analyzed a separate cohort of 58 patients who received CROSS neoadjuvant therapy followed by surgery and adjuvant Nivolumab therapy for 1 year, of whom survival data was available for 40 patients. This cohort had distinct baseline characteristics compared to the primary cohort (**Table 3**). Using the same median-based dichotomization approach, patients were stratified into MECA-low and MECA-high (both n=20) groups.

**Table 3:** Baseline characteristics of 58 patients who received adjuvant Nivolumab therapy.

Characteristic	N=58
Age at surgery — mean ± SD	62.2 (8.7)
Sex — no. (%)	
Male	49
Female	9
Tumor stage (T) — no. (%)	
T0	4 (6.9)
T1	11 (19.0)
T2	11 (19.0)
T3	31 (53.4)
T4	1 (1.7)
Nodal stage (N) — no. (%)	
N0	30 (51.7)
N1	16 (27.6)
N2	10 (17.2)
N3	2 (3.4)

**Figure 5:**
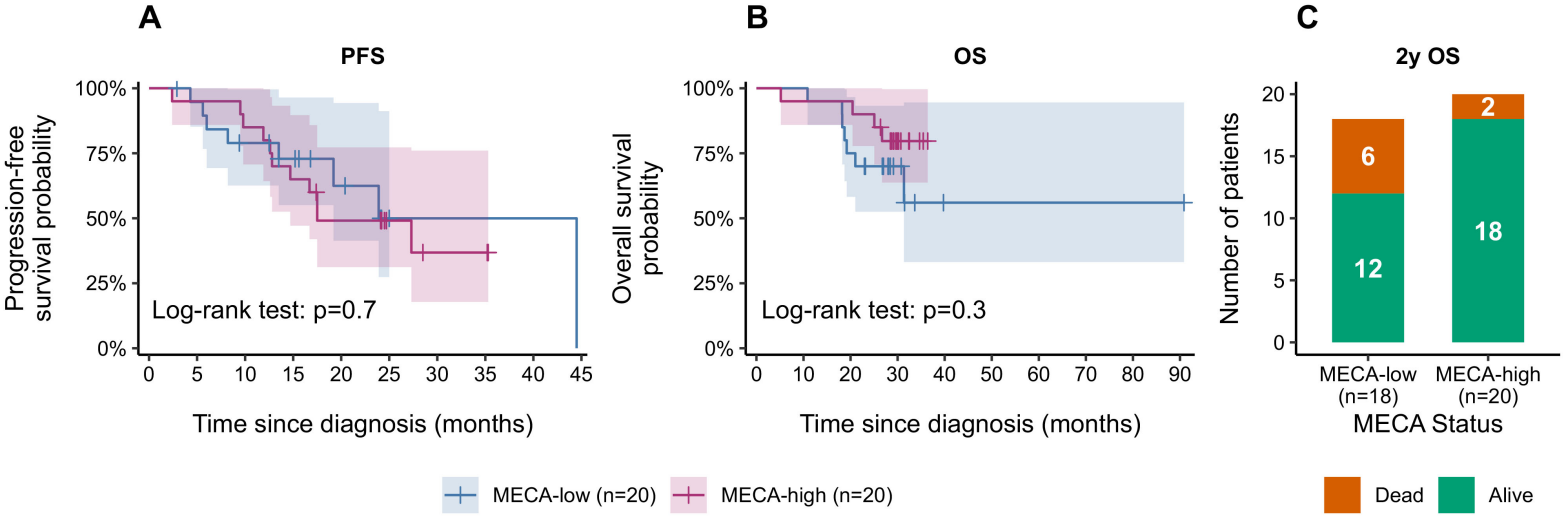
Survival analysis in the adjuvant Nivolumab cohort stratified by HEV density. **(A)** Progression-free survival curves for MECA-low and MECA-high groups (log-rank test: p=0.7). **(B)** Overall survival curves for MECA-low and MECA-high groups (log-rank test: p=0.3). **(C)** Two-year overall survival analysis showing number of patients alive (green) and dead (orange) in each group. MECA-low: 66.7% survival (12/18); MECA-high: 90.0% survival (18/20) (Fisher’s exact test: p=0.1171).

Survival analysis in this immunotherapy cohort showed patterns consistent with the primary cohort (**Figure 5**). Overall survival demonstrated no significant difference between MECA groups (p=0.2633), though a trend toward improved survival in the MECA-high group was observed. Progression-free survival analysis showed similar curves between groups, indicating no meaningful difference. Two-year overall survival analysis revealed that 18 of 20 patients (90%) in the MECA-high group remained alive compared to 12 of 18 patients (67%) in the MECA-low group, though this difference did not reach statistical significance (p=0.1171, Fisher’s exact test). These findings are consistent with the trend observed in the primary cohort, where MECA-high patients also showed a tendency toward better survival outcomes, suggesting that higher HEV density may be associated with improved prognosis across different treatment contexts.

## Discussion

In this study, we provide a systematic characterization of high endothelial venules (HEVs) in tumor-free regional lymph nodes of patients with esophageal adenocarcinoma (EAC) and evaluate their relationship with clinicopathological features, treatment modality, age, and survival outcomes. While HEVs have previously been associated with favorable prognosis and enhanced anti-tumor immunity in esophageal squamous cell carcinoma and gastric cancer, their role in EAC—particularly within regional lymph nodes—has remained undefined. Our data reveal a pronounced age-dependent increase in lymph node HEV density that is largely independent of tumor stage, nodal status, and response to neoadjuvant therapy, and only weakly associated with survival outcomes. These findings suggest that HEV density in regional lymph nodes reflects host-related immunological factors rather than tumor-intrinsic aggressiveness or treatment efficacy in EAC.

A central and unexpected finding of our study is that patient age emerged as the dominant determinant of HEV density, outweighing the influence of neoadjuvant treatment modality, pathological response, or TNM stage. Across treatment groups, patients older than 60 years consistently exhibited higher HEV densities, with this association reaching statistical significance particularly in the CROSS-treated cohort. Importantly, this age-related pattern persisted regardless of therapy response, indicating that HEV abundance in regional lymph nodes is not merely a surrogate of treatment-induced immune activation.

At first glance, the observation of increased HEV density with advancing age may appear counterintuitive, given the well-established concept of immunosenescence, which is typically characterized by diminished naïve T-cell output, reduced lymphoid tissue function, and impaired adaptive immune responses (10, 11). However, accumulating evidence suggests that aging is also associated with chronic low-grade inflammation (“inflammaging”), lymphoid tissue remodeling, and compensatory vascular and stromal adaptations within secondary lymphoid organs. Experimental studies have demonstrated age-related alterations in lymph node architecture, including expansion of stromal networks and endothelial specialization, potentially facilitating enhanced lymphocyte trafficking despite reduced immune repertoire diversity (13). Our findings are consistent with this paradigm and suggest that HEV density in regional lymph nodes may represent a structural adaptation to long-term antigenic exposure rather than a marker of effective anti-tumor immunity per se.

Notably, HEV density was not associated with pathological response to neoadjuvant therapy, either in FLOT- or CROSS-treated patients. This contrasts with prior reports in other tumor entities where therapy-induced HEV formation—particularly following immune checkpoint blockade or anti-angiogenic treatment—has been linked to improved immune infiltration and tumor regression. Several factors may explain this discrepancy. First, our analysis focused exclusively on tumor-free regional lymph nodes, rather than intratumoral or peritumoral HEVs, which are more directly involved in effector T-cell recruitment to the tumor microenvironment. Second, cytotoxic chemotherapy and chemoradiotherapy may exert divergent effects on lymphoid stromal compartments compared to immunomodulatory agents, potentially limiting their capacity to induce functional HEV neogenesis. Finally, the lack of association with therapy response underscores that lymph node HEV density reflects a pre-existing or host-conditioned immune microanatomy, rather than a dynamic marker of treatment sensitivity.

An important consideration when interpreting our findings is the spatial heterogeneity of HEVs across distinct anatomical compartments, each of which may carry different functional implications. HEVs in regional lymph nodes (the focus of our study) represent the homeostatic vascular gateway for lymphocyte trafficking, but in tumor-draining lymph nodes they are increasingly recognized to undergo “dedifferentiation” through tumor-secreted factors such as VEGFD, with consequent impairment of lymphocyte homing (18). In contrast, intratumoral HEVs (TA-HEVs) form *de novo* within tumors, mediate effector lymphocyte entry, and predict response to combined PD-1/CTLA-4 blockade in melanoma and other solid tumors (2, 16). A third compartment comprises HEVs embedded within tertiary lymphoid structures, which serve as local immune hubs and have recently been shown by single-cell and spatial transcriptomics to be specifically enriched as SELP+ACKR1+ HEV cells in intratumoral-TLS-rich gastric cancers (17, 19). Notably, recent single-cell evidence in ESCC indicates that neoadjuvant chemoimmunotherapy expands the venous endothelial compartment from which HEVs derive (8), with intratumoral and invasive-margin TLS abundance further linked to improved overall survival in resectable ESCC under this regimen (20); in addition, Fc-engineered checkpoint inhibitors can therapeutically induce TA-HEV formation in preclinical models (4). Our cohort differs from these settings in three respects: EAC rather than ESCC, melanoma, or gastric cancer; regional lymph node rather than tumor tissue; and chemotherapy or chemoradiotherapy rather than immunotherapy—caveats that may also explain the trend toward better outcomes in MECA-high patients receiving adjuvant nivolumab.

Consistent with this interpretation, HEV density showed no correlation with T-stage, N-stage, or overall pathological tumor burden, reinforcing the notion that HEVs in regional lymph nodes are decoupled from tumor progression in EAC. This finding is particularly relevant given that lymph nodes represent both key sites of immune priming and common reservoirs for early metastatic spread. While HEVs within tertiary lymphoid structures in primary tumors have been repeatedly linked to favorable prognosis in other gastrointestinal malignancies (1), our data indicate that lymph node HEVs alone are insufficient to capture clinically meaningful anti-tumor immune activity in EAC.

Survival analyses further support this conclusion. Despite a consistent trend toward improved outcomes in MECA-high patients, HEV density did not significantly predict overall survival or progression-free survival in either the primary cohort or the adjuvant nivolumab-treated subgroup. Importantly, this absence of statistical significance should not be interpreted as evidence against a biological role of HEVs, but rather reflects the complexity of immune–tumor interactions in EAC and the multifactorial determinants of survival. The numerically higher two-year overall survival observed in MECA-high patients receiving adjuvant nivolumab suggests that HEV-rich lymph nodes may facilitate more effective immune reconstitution or T-cell priming in the context of checkpoint blockade. However, the limited sample size and retrospective nature of this subgroup analysis preclude definitive conclusions.

From a translational perspective, our findings suggest that HEV density in regional lymph nodes is unlikely to serve as a standalone prognostic biomarker in unselected EAC patients, but may still hold value as part of a composite immune signature. Integrating HEV quantification with additional parameters—such as lymphocyte subset composition, spatial immune profiling, or intratumoral tertiary lymphoid structure assessment—may improve its predictive relevance, particularly in the setting of immunotherapy. Moreover, the observed age dependency highlights the need to account for host-related immunological factors when developing and validating immune biomarkers in EAC.

Several limitations of this study merit consideration. Its retrospective design and single-center nature may limit generalizability, and survival analyses—especially in the immunotherapy cohort—were constrained by sample size and follow-up duration. Additionally, functional characterization of HEVs and their associated immune cell populations was beyond the scope of this work. Future studies incorporating multiplex immunohistochemistry, spatial transcriptomics, or single-cell analyses will be essential to elucidate whether lymph node HEVs in older patients are functionally competent or represent structurally preserved but immunologically impaired vessels. Furthermore, our analysis was confined to regional lymph nodes; paired profiling of intratumoral HEVs and tertiary lymphoid structures was not performed and would be required to formally relate compartment-specific HEV biology to treatment response.

In conclusion, HEV density in tumor-free regional lymph nodes of EAC patients is primarily age-dependent and independent of tumor stage, treatment modality, and pathological response. Although it does not by itself predict survival, its potential interaction with immunotherapy response warrants further validation in larger cohorts and underscores the importance of accounting for host-related immune architecture when developing lymph node–based immune biomarkers in EAC.

## Data Availability

The raw data supporting the conclusions of this article will be made available by the authors, without undue reservation.

## Supplementary material

Supplementary Figure 1 HEV density distribution across TNM staging categories. **(A)** HEV density by T-stage (T0-T4). **(B)** HEV density by N-stage (N0-N3). Data presented as violin plots with boxplots and individual data points. Statistical comparisons performed using Kruskal-Wallis tests (T-stage: p=0.971, N-stage: p=0.912).
