# Supplementary material for: Clinical and prognostic significance of high-endothelial venule density in regional lymph nodes of esophageal adenocarcinoma
Source: Front Oncol. 2026 Jul 14;16:1802849. doi: 10.3389/fonc.2026.1802849 (PMC13407108; doi:10.3389/fonc.2026.1802849)
Supplement: Supplementary Figure 1 — HEV density distribution across TNM staging categories. (A) HEV density by T-stage (T0-T4). (B) HEV density by N-stage (N0-N3). Data presented as violin plots with boxplots and individual data points. Statistical comparisons performed using Kruskal-Wallis tests (T-stage: p=0.971, N-stage: p=0.912). [file Supplementaryfile1.docx]

**Supplementary Materials** **for:**

**Clinical and prognostic significance of high-endothelial venule density in regional lymph nodes of esophageal adenocarcinoma**

Aryan Mirzaie-Kuzehgarani, Tillmann Bedau, Johanna Teloh-Benger, Thomas Zander, Hans Anton Schlößer, Reinhard Büttner, Christiane Bruns, Alexander Quaas

**Table of Contents**

[Supplementary Figures 2](#_Toc233650966)

[Supplementary Figure S1 2](#_Toc233650967)

## Supplementary Figures

### Supplementary Figure S1


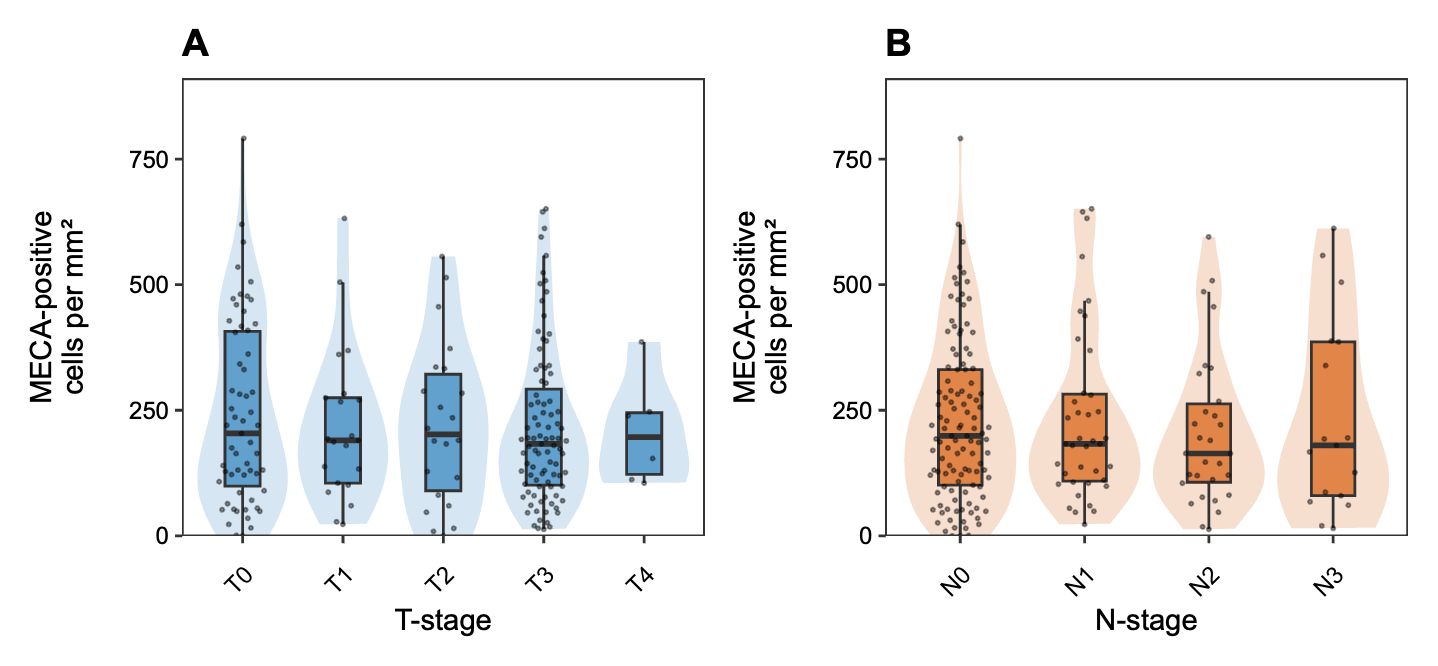
 **HEV density distribution across TNM staging categories. (A)** HEV density by T-stage (T0-T4). **(B)** HEV density by N-stage (N0-N3). Data presented as violin plots with boxplots and individual data points. Statistical comparisons performed using Kruskal-Wallis tests (T-stage: p=0.971, N-stage: p=0.912).
